# Complete genome sequence of *Pseudomonas* phage Ep4 lysing the kiwifruit canker bacteria

**DOI:** 10.1128/mra.00947-23

**Published:** 2023-12-19

**Authors:** Mitsuo Aono, Haruka Yagi, Kappei Kobayashi

**Affiliations:** 1 Fruit Tree Research Center, Ehime Research Institute of Agriculture, Forestry and Fisheries, Matsuyama, Ehime, Japan; 2 The United Graduate School of Agricultural Sciences, Ehime University, Matsuyama, Ehime, Japan; The University of Arizona, Tucson, Arizona, USA

**Keywords:** bacteriophage, kiwifruit canker, genome sequence

## Abstract

*Pseudomonas syringae* pv. *actinidiae* is a pathogen of kiwifruit canker. Ep4, a bacteriophage lysing the pathogenic bacteria, was isolated from an affected plant. Sequencing and annotation have revealed 44,614-bp genome with 52 predicted open reading frames. Ep4 is closest to *Pseudomonas* phage YMC11/06/C171_PPU_BP, albeit with low homology.

## ANNOUNCEMENT

*Pseudomonas syringae* pv. *actinidiae* (Psa) causes bacterial canker of kiwifruits with reddish brown exudate, shoot dieback, brown leaf spots, etc. (1). Studies have classified Psa into five biovars based on multilocus sequence analysis (2). Among them, four biovars, except for biovar 2, were confirmed in Japan (2). We searched for bacteriophages lysing Psa to develop biocontrol agents and isolated *Pseudomonas* phage Ep4 from a lesion in a leader (lateral branch) of a diseased kiwifruit in Matsuyama, Ehime, Japan (33°54′52.4″N 132°47′40.6″E). The phage was isolated, as essentially described previously (3), with slight modifications. Briefly, the sap of a lesion was mixed with 5 mL of Nutrient Broth (Difco BD, NJ, USA) and cultured overnight at 25°C. After centrifugation (12,000 × *g*, 10 min), the supernatant was subjected to phage isolation by the soft agar overlay method (4) using Psa biovar 1 (MAFF211985) as a host.

The DNA was extracted with a Phage DNA Isolation Kit (Norgen Biotek, ON, Canada) according to the manufacturer’s instructions. The sequencing library was prepared using Illumina DNA Prep (M) Tagmentation (Illumina, CA, USA), and the entire genome was sequenced on the Illumina MiSeq with paired-end 156-bp reads using V2 300-cycle chemistry by Genome-Lead Corporation (Kagawa, Japan). A total of 319,572 reads were quality checked by FastQC v.0.72 (5), trimmed using the Trimmomatic v.0.38.0 (6), and assembled to a single raw contig at 35.0-fold coverage using Shovill v.1.1.0 (7) with default parameters on Galaxy (https://usegalaxy.org).

The BLASTn and PHASTER (8) searches of the assembled contig revealed that Ep4 was closest to *Pseudomonas* phage YMC11/06/C171_PPU_BP (NCBI Reference Sequence: NC_030923.1) of *Autographiviridae*. The terminal region of the genome was estimated by DTR sequences of some *Autographiviridae* and the starting position coverage of PhageTerm (9) on Galaxy Pasteur (https://galaxy.pasteur.fr). The terminal sequences were determined by sequencing PCR fragments amplified from the genome DNA template after adding G-tails using terminal deoxynucleotidyl transferase using different genome-specific primers and a primer binding to the G-tails (5′-CGCTCTAGAACTAGTGGATCCCCCCCCCCCCD-3′). The assembled contig was reopened manually using GENETYX ver.15 (Genetyx, Tokyo, Japan) based on the terminal sequences.

Ep4 genome was 44,614 bp in length, containing 264 bp of direct terminal repeats and 56.0% GC content. Open reading frames (ORFs) were predicted with RAST v.2.0 (https://rast.nmpdr.org), PHANOTATE (10) on CPT Galaxy (https://cpt.tamu.edu/galaxy-pub), and GeneMarkS (11). Out of the predicted 52 ORFs, 32 were assigned putative functions by the BLASTp on the NCBI database with an *E* value less than 1 × 10^−5^. Ep4 had proteins related to structure, host lysis, and DNA replication. The search for tRNA sequences using ARAGORN (12) and tRNAscan-SE 2.0 (13) showed no tRNA genes.

**Fig 1:**
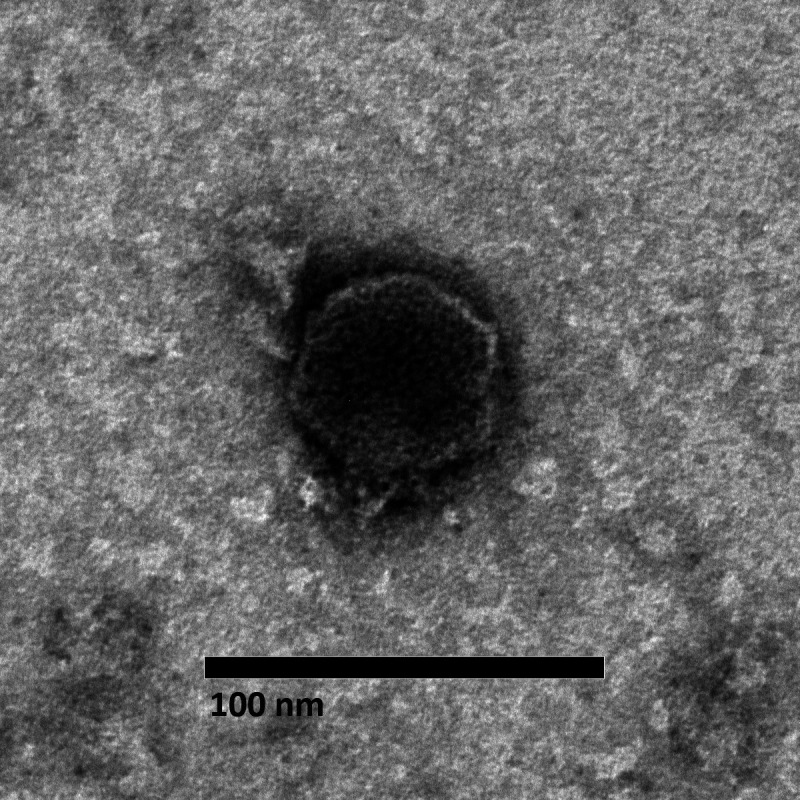
Electron micrograph of Ep4. Purified phage was spotted onto carbon-film-coated transmission electron microscopy grids, negatively stained with 2% uranyl acetate, and analyzed using an H-7600 transmission electron microscope (Hitachi, Tokyo, Japan) by the Hanaichi UltraStructure Research Institute (Aichi, Japan).

Morphological analysis revealed short-tailed particles like those of *Autographiviridae* (Fig. 1). Phylogenetic analysis based on putative terminase large subunit showed the closest relationship with YMC11/06/C171_PPU_BP. The relationship between YMC11/06/C171_PPU_BP and Ep4 was supported by a proteome-wide search in the AAI-profiler (14). However, the amino acid identity value between YMC11/06/C171_PPU_BP and Ep4 was as low as 0.669.

## Data Availability

The genome sequence and associated data for Ep4 have been deposited to DDBJ under accession number LC776701, BioProject accession number PRJDB16165, SRA accession number DRR491366, and BioSample accession number SAMD00630467 .
